# Long-term dietary restriction changes lipid homeostasis and consequently impairs testosterone production in aged *Wistar* rats

**DOI:** 10.3389/fnut.2025.1665682

**Published:** 2025-09-19

**Authors:** Srdjan Sokanovic, Milica Prvulovic, Valentina Simeunovic, Smilja Pracer, Aleksandra Mladenovic

**Affiliations:** Laboratory for Molecular Neurobiology and Behavior, Department of Neurobiology, Institute for Biological Research “Sinisa Stankovic”—National Institute of the Republic of Serbia, University of Belgrade, Belgrade, Serbia

**Keywords:** testosterone, cholesterol, LDL-C, HDL-C, long-term dietary restriction, short-term dietary restriction

## Abstract

Testosterone (T) is a central androgen responsible for the maintenance of vegetative and reproductive functions and sexual behavior in males. T is mainly synthesized through the process of testicular steroidogenesis with cholesterol (CHOL) as the initial precursor. It is known that T levels gradually decrease, along with an increase in CHOL, LDL-C, triglycerides (TG), and a decrease in HDL-C in advanced stages of life. Dietary restriction (DR) ameliorates lipid status and raises T levels in obese and overweight men. Here, we investigated whether the beneficial effects of DR on serum lipid status consequently improve T production at advanced stages; therefore, we exposed male *Wistar rats* to long-term DR (LTDR, 18 months). We confirmed an age-related decrease in serum T levels, reduced expression of genes and proteins of steroidogenic machinery with a simultaneous increase in serum CHOL, LDL-C, and TG levels. LTDR additionally decreased T synthesis, expression of *Star*/StAR and *Cyp11a1*/CYP11A1, and testicular CHOL levels. At the same time, LTDR reduced serum CHOL, LDL-C, and TG levels and increased HDL-C levels. To confirm that the effects of DR are determined by the duration of the treatment, we also checked the effects of the short-term DR (STDR, 3 months) and demonstrated that STDR did not alter T levels and did not affect serum lipids. Our results indicate the importance of sustained systemic lipid homeostasis for T production in advanced life stages and show that the effects of restricted food intake on testicular androgen capacity depend on the duration of DR.

## 1 Introduction

Testosterone (T) is a pivotal male androgen responsible for maintenance of numerous physiological functions including spermatogenesis, secondary sexual characteristics (1) as well as sexual behavior (2). T is primarily synthesized and produced by testes Leydig cells (LCs) through the complex process of steroidogenesis, which involves enzymatic transformation of cholesterol (CHOL) to T. Steroidogenic cells take up CHOL from circulating high- and low-density lipoproteins (HDL-C, LDL-C) (3), or synthesize it *de novo* (4). In rat Leydig cells, the inner leaflet of the plasma membrane also represents a source of CHOL for steroidogenesis (5). The dominant source of CHOL may be species specific, but it is widely accepted that HDL-C represents the main extracellular source of CHOL for steroidogenesis in rodents. The process is mediated by membrane scavenger receptor B1 (SR-B1, SCARB1) (6). In the cells, lysosomal acid lipase (LAL), hormone-sensitive neutral lipase (HSL), together with Niemann–Pick disease type C1 and 2 (NPC1/2) proteins, account for the formation of free CHOL, making it available for steroidogenesis or (re)esterification by acyl-coenzyme A-cholesterol acyltransferase activity (ACAT/SOAT1) (7). During the initial steps of steroidogenesis, a complex protein machinery and its key component, steroidogenic acute regulatory protein (StAR) facilitates import of free CHOL into mitochondria (8, 9) where cytochrome P450 side chain cleavage enzyme (P450scc, CYP11A1) catalyzes formation of pregnenolone. Subsequent reactions occur in the endoplasmic reticulum, where 3 beta-hydroxysteroid dehydrogenase (HSD3B) converts pregnenolone to progesterone, and cytochrome P450 family 17 subfamily A member 1 (CYP17A1) transforms progesterone into androstenedione. In the final reaction, 17 beta-hydroxysteroid dehydrogenase (HSD17B) transforms androstenedione to T. The described pathway, known as the Δ^4^ steroidogenic pathway, is dominant in rodents (10). Testicular steroidogenesis is primarily regulated by the hypothalamic-pituitary-testicular loop and luteinizing hormone-cyclic adenosine monophosphate (LH-cAMP) signaling pathway. In this cascade, hypothalamic gonadotropin-releasing hormone (GnRH) stimulates pituitary gonadotrophs to synthesize and release luteinizing hormone (LH) (11), which activates LH receptors (LHR) expressed on the surface of LCs. Activation of the receptors promotes a rise in cAMP, which initiates steroidogenesis (12) and inhibits SOAT1 activity, making CHOL available for T synthesis (6). Increased circulation levels of T inhibit the loop activity and suppress its production.

Aging is a complex process associated with failure in multiple organ systems (13), including changes in T levels and CHOL homeostasis, and a gradual decline in T levels in both humans (14) and rats (15, 16) has been demonstrated. Plenty of data indicate that age-induced impairment in T production is a consequence of impaired functionality of LCs (17) rather than alteration in LCs numbers (18). Mechanisms responsible for the reduced functionality of aged LCs are far from elucidated, but it is known that aged LCs do not respond properly to LH (17, 19), show decreased expression and activity of steroidogenic enzymes (17), and weak expression of Insulin-like 3 peptide, its functionality marker (INSL3) (15). Regarding age-induced alterations in lipid homeostasis, increased levels of total CHOL, LDL-C, and decreased HDL-C have been documented in humans (20). Elevated levels of triglyceride (TG) and CHOL (21, 22) with reduced clearance of LDL-C (23) have been documented in rats. Advanced age is also accompanied by a frequent appearance of dyslipidemia, a condition characterized by increased levels of plasma TG and LDL-C with decreased levels of HDL-C, which represents major factors for the development of coronary artery disease (CAD) and cardiovascular diseases (CVD) (20).

Low T levels are associated with both sexual and non-sexual symptoms, which profoundly impact the quality of men’s lives. Non-sexual symptoms associated with low T concentrations include the appearance of obesity, type 2 diabetes mellitus, metabolic syndrome, etc. (24). In the context of low T levels and disrupted lipid homeostasis, there is a documented association between sub-physiological levels of T and atherogenic lipid formation, which promotes pathogenesis of atherosclerosis and CAD (25, 26). Parameter that is highly correlated with low T levels and CAD/CVD appearance is a high TG to HDL-C (TG/HDL-C) ratio. A high TG/HLD-C ratio has been proposed as a suitable parameter for CAD/CVD prediction (27), and it is also shown that TG/HLD-C ratio is inversely correlated with testosterone levels in the case of insulin resistance (28).

Dietary restriction (DR) is the most effective non-pharmacological intervention that enhances longevity and health span in numerous nonhuman species and generally refers to the reduction of calories without malnutrition (29). Numerous animal and clinical studies suggest that DR positively affects age-related neurodegenerative diseases (29), animal activity and spatial memory (30), metabolic disorders (31, 32), decreased systolic and diastolic blood pressure, and has a protective effect against atherosclerosis through decrement of total CHOL, LDL-C, TG and increment of HDL-C (33). DR showed dual effects on T production with beneficial effects in obese and overweight men, and negative effects in non-obese men (24) and rats (34–36). To examine the way DR impacts testicular steroidogenesis in advanced periods of life, we exposed male *Wistar* rats to 18- and 3-month-long DR. The effects of the treatments were assessed based on the T levels in circulation and testicular content, with concomitant lipid status profile. Molecular mechanisms were evaluated based on the expression of the genes and proteins involved in T synthesis as well as CHOL metabolism.

## 2 Materials and methods

### 2.1 Animals and treatments

Male *Wistar* rats were used in this study (*n* > 30, 5–10 animals per group). All animal handling procedures complied with the EU Directive 2010/63/EU for animal experiments and were approved by the Ethical Committee for the Use of Laboratory Animals of the Institute for Biological Research “Sinisa Stankovic”—The National Institute of the Republic of Serbia, University of Belgrade, and by The National Ethic Research Committee (No. 323-07-13, No. 536/2020-05, No. 05-06/14). Animals were housed in a 12-h light/dark cycle with constant temperature (21 ± 2 °C) and humidity (60–70%). Animals were fed with standard laboratory chow (chemical composition presented in the Table 1, manufacturer VZS Stocna hrana d.o.o, Subotica, Serbia) with free access to water. DR treatment used in this study represents a reduction of daily food intake by 40% based on average daily food consumption. This study included two DR regimes: long-term DR (LTDR), started at 6 months and lasted up to the 24th month of life (*n* = 10), and short-term DR (STDR), started at 21 months and lasted up to the 24th month of life (*n* = 10); the experiments were repeated two times. Intact 24-month-old males (aged, *n* = 10) with no limited access to food were used as controls for LTDR and STDR, the study also included intact 6-month-old males (adults, *n* = 5). Animals were regularly weighed and monitored for well-being during the entire study period. At the end of the treatments, animals were euthanized in deep anesthesia using Zoletil 100 (75 mg/kg), blood was collected from the heart, and transcardial perfusion with cold saline was performed, and tissues were collected and stored at −80 °C. The serum was prepared by centrifuging coagulated blood (300 × g/15 min/4 °C) and stored at −20 °C until analyzing.

**Table 1:** Chemical composition of standard laboratory chow.

Ingredient	Quantity	Ingredient	Quantity
Proteins	20%	Vitamin A	12,000 U/kg
Fat	5%	Vitamin D3	2,000 U/kg
Humidity	11.5%	Vitamin E	30 mg/kg
Cellulose	5%	Vitamin B1	4 mg/kg
Ashes	8%	Vitamin B2	7 mg/kg
Lysine	0.6%	Vitamin B6	6 mg/kg
Methionine	0.6%	Vitamin B12	0.05 mg/kg
Tryptophan	0.15%	Folic acid	1 mg/kg
Calcium	0.95%	Choline chloride	1,000 mg/kg
Phosphorus	0.7%	Niacin	20 mg/kg
Zink	30 mg/kg	Pantothenic acid	10 mg/kg
Copper	15 mg/kg	Antioxidants E-202	125 mg/kg
Iron	60 mg/kg	Antioxidants E-320	
Magnesium	60 mg/kg	Antioxidants E-321	
Iodine	0.15 mg/kg	Antioxidants E-338	
Selenium	0.3 mg/kg		

### 2.2 Serum lipid measurements

To assess serum lipid status, concentrations of CHOL, HDL-C, LDL-C, and TG were measured. All the reagents and standards were obtained from the same manufacturer (BioSystems S.A. Costa Brava, Barcelona, Spain), and measurements were performed according to the manufacturer’s instructions^1^ using a clinical chemistry analyzer BS-240 (Mindray, Shenzhen, China). Detection limits were: 0.008 mmoL/L for CHOL, 0.01 mmoL/L for HDL-C, 0.007 mmoL/L for LDL-C, 0.018 mmoL/L for TG; for all the parameters, intra/inter run variations were <5%.

### 2.3 Hormone assays and steroid extraction

Serum hormone levels were determined with appropriate ELISA kits according to the manufacturer’s instructions. For corticosterone measurement, we used Enzo corticosterone ELISA kit (ADI-900-097, Enzo Life Science, Farmingtone, NY, United States), and for T measurement, we used Cayman Testosterone ELISA kit (No. 582701, Cayman Chemical, Ann Arbor, MI, United States). To determine T levels in testes, pieces of testes were homogenized with 1 × PBS (137 mM NaCl, 2.7 mM KCl, 10 mM Na_2_HPO_4_, 1.8 mM KH_2_PO_4_, v/w = 10 μL/mg) and an equal volume of homogenate was used for steroid extraction by diethyl ether. After evaporation of diethyl ether, the dry pellet was resuspended with ELISA buffer, and T levels were determined by the same ELISA kit (15). All the relevant comparisons were made in the same assay; intra-assay variation for corticosterone was <10% and for T was <15%; minimal detectable hormone concentrations were 32 pg/mL and 3.9 pg/mL, respectively. To determine levels of testicular CHOL, the same procedure of extraction was applied, the dry pellet was resuspended in 1 × PBS enriched with bovine serum albumin fraction V (1 × PBS-0.1%-BSA), and CHOL levels were determined according to Biosystems protocol (see text footnote 1) and BS-240 analyzer (Mindray, Shenzhen, China).

### 2.4 RNA isolation and qRT-PCR analysis

Total RNA was isolated from pieces of testes, liver, and whole pituitary by Trizol reagent (300 μL/sample, Ambion, Austin, TX) with the addition of chloroform (60 μL/sample). After shaking and centrifugation (12,000 × g/15 min/4 °C) supernatant was collected, RNA was extracted by isopropyl alcohol (250 μL/sample) and centrifugation (12,000 × g/10 min/4 °C). Collected RNA was washed twice with 75% ethanol, resuspended in RNase-free water, and DNase I treatment was performed according to Invitrogen manufacturer’s protocol.^2^ The amount and purity of the total RNA were assessed by spectrophotometric measurement (Nanophotometer N60, Implen, Munchen, Germany), and 1 mg of RNA was used for cDNA synthesis. First-strand cDNA was synthesized by High-Capacity cDNA reverse transcription kit^3^ according to the manufacturer’s instructions. qPCR was performed by Taq-Man Gene- or SYBR Green-based technology (Applied Biotechnology, Waltham, Massachusetts, United States) in the presence of 10 ng cDNA using Quant Studio 3 (Applied Biotechnology). Target gene expression levels were determined by the comparative 2^−ΔΔCT^ quantification method with *Gapdh* as the reference gene. Used Applied Biosystems predesigned TaqMan Gene Expression Assays: *Abca1* (Rn00710172_m1), *Apob* (01499054_m1), Cyp46a1 (Rn01430187_g1), *Gapdh* (Rn01462662_g1), *Hmgc* (Rn 00565598_m1), *Lrp1* (Rn01503901), *Ldlr* (Rn00598442_m1), *Npc1* (Rn01531821_m1), *Soat1* (Rn_00579605_m1), *Srebf1* (Rn01495769_m1); sequences of used forward and reverse primers are listed in Supplementary Table 1.

### 2.5 Protein isolation and western blot method

Proteins were isolated from the pieces of testes and whole pituitary tissues using Ripa buffer (50 mM Tris-HCl, 150 mM NaCl, 1% NP-40, 0.1% SDS, 10 mM EDTA, 10 mM EGTA, 0.5% Triton X-100; V/W = 10 μL/mg) enriched with protease and phosphatase inhibitor cocktails (Roche, GmbH, Germany). After homogenization, the samples were centrifuged (15,000 × g/30 min/4 °C), supernatants were collected, and protein concentration measurement and uniformization of the samples were done by Pierce BSA Protein Assay Kit (ThermoFisher Scientific, Waltham, MA, United States). An equal amount of protein (50 μg/well) was loaded onto 10–15% SDS-polyacrylamide gels and separated by SDS-PAGE electrophoresis (Bio-Rad, Hercules, CA, United States). Transfer of proteins onto nitrocellulose (0.45 μm, Amersham, MA, United States) or PVDF (0.2 μm, Invitrogen, Waltham, MA, United States) membrane was done by wet transfer (30 V/4 °C, overnight) using Bio-Rad system. Membranes were stained with 0.1% Ponceau S (Serva Electrophoresis GmbH, Germany) or Pierce Reversible Protein Stain Kit for nitrocellulose membrane (ThermoFisher Scientific), scanned, and used as the loading control. Destaining was done by 0.1% Tween-TBS (0.05 M Tris, 0.15 M NaCl, 0.1% Twen; TBST) following membrane blocking with 5% non-fat dry milk (Bio-Rad) in TBST for 2 h at room temperature (RT). Membranes were incubated with primary antibodies overnight at 4 °C, multiple washed with TBST, and incubated with horseradish peroxidase conjugated secondary anti-rabbit antibody for 1 h/RT. After sufficient washing, chemiluminescent signal was induced by Luminol-H_2_O_2_ solution, and immunoblots were visualized using iBright 1500 system (Invitrogen). Quantification of the specific immunoblots, as well as loading control, was performed by Image quant software (version 5.2, GE Healthcare). Used antibodies with dilutions: anti-CYP11A1 antibody (13363-1-AP, Proteintech, Rosemont, IL, United States) 1:1000; anti-LhB antibody (PA5-102674, ThermoFisher Scientific) 1:1,000; anti-StAR antibody (D0H12, Cell Signaling Technology Inc., MA, United States) 1:500; Goat anti-Rabbit antibody (ab205718, Cambridge, United Kingdom) 1:10,000.

### 2.6 Statistical analysis

Results represent group mean ± SEM values of at least 5 animals per group. Multiple comparison groups were analyzed by one-way ANOVA followed by Tukey’s multiple comparison test; comparison between two groups was analyzed by Student’s *t*-test or unpaired Mann–Whitney nonparametric two-tailed tests; *p* < 0.05 was considered statistically significant. GraphPad Prism software (version 6, Dotmatics, MA, United States) was used for mean ± SEM values calculations, statistical analysis, and figure generation.

## 3 Results

### 3.1 LTDR changes BW, lipid status, and T levels in aged male *Wistar* rats

**Figure 1:**
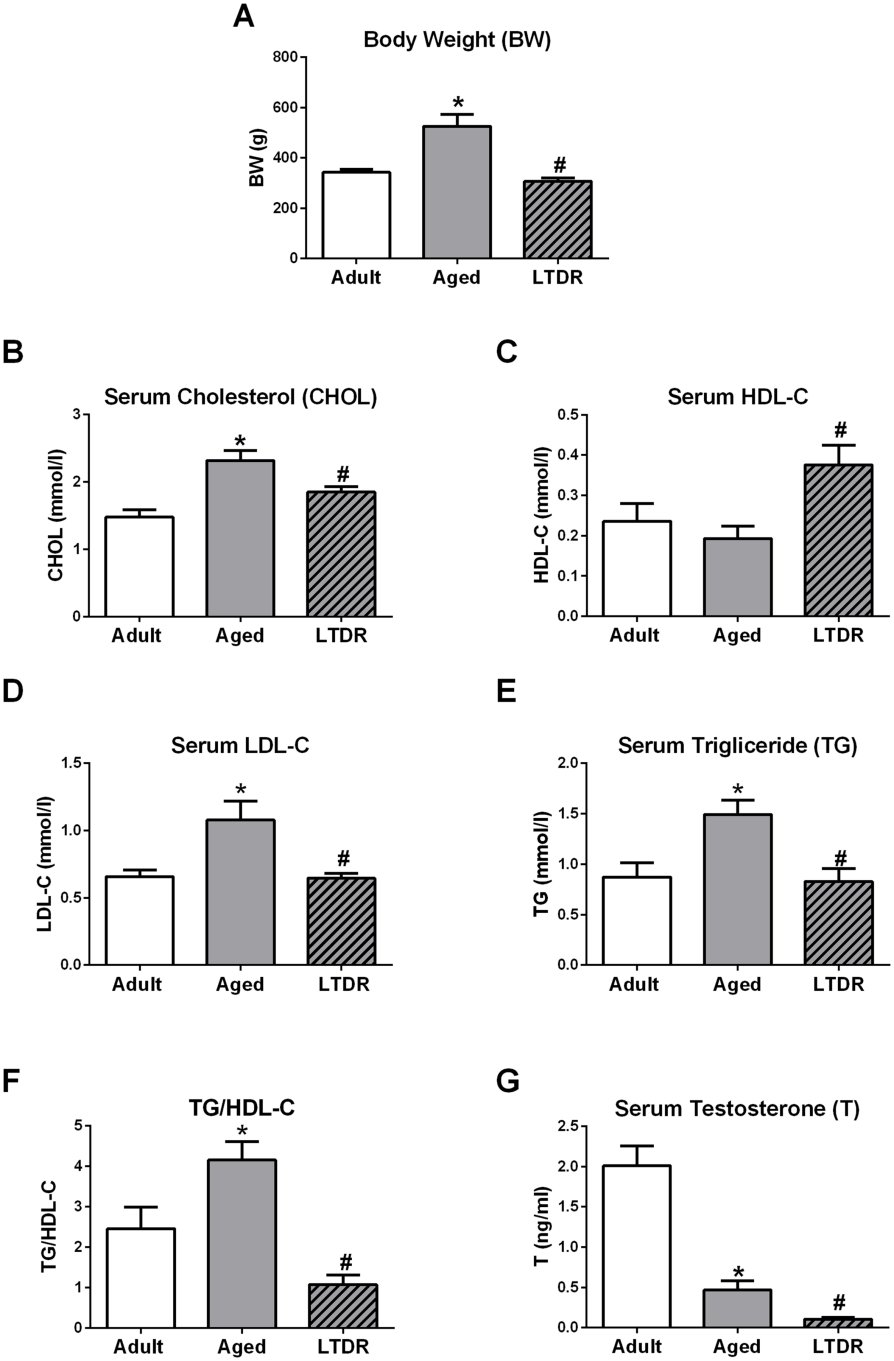
Effects of the aging and LTDR on the males BW, lipids profile and T levels. 6-month-old (adults), 24-month-old male *Wistar* rats (aged) and males subjected to long-term dietary restriction (18-month-long, 60% of daily food intake, LTDR) were followed to estimate time- and LTDR-induced changes in BW **(A)**. Serum from all the groups was prepared and lipid status was characterized by photometric measurements of CHOL **(B)**, HDL-C **(C)**, LDL-C **(D)** and TG **(E)** levels, and TG/HDL-C ratio was calculated **(F)**. T was determined by ELISA method revealing age induced decrement in T levels with deterioration after LTDR regime **(G)**. The bars shown are mean ± SEM values; for all the analysis *n* ≥ 5; ^*^indicates significant differences compared to adults; ^#^indicates significant differences between aged animals and the LTDR group; *p* < 0.05.

As BW, T levels, and lipid homeostasis are tightly connected, we first checked how aging and LTDR affect these parameters. Comparing 6-month-old (adult) and aged males, we observed that aging is accompanied with significant BW gain (adults: 342.5 ± 12.64 g; aged: 525 ± 47.87 g), and that LTDR abolished this effect (LTDR: 306.0 ± 14.70 g; aged: 525 ± 47.87 g) preserving BW to the values similar to the BW of adults (Figure 1A). Analyses of lipid status revealed significant alterations in aged animals in comparison to adults: elevated serum levels of CHOL (adults: 1.48 ± 0.10 mmoL/L; aged: 2.32 ± 0.15 mmoL/L), LDL-C (adults: 0.65 ± 0.05 mmoL/L; aged: 1.05 ± 0.14 mmoL/L), TG (adults: 0.87 ± 0.14 mmoL/L; aged: 1.49 ± 0.14 mmoL/L) and TG/HDL-C ratio (adult: 2.45 ± 0.54; aged: 4.15 ± 0.45). In all cases, LTDR effectively normalized levels of examined parameters to the levels detected in adults (CHOL: 1.85 ± 0.08 mmoL/L; LDL-C: 0.64 ± 0.03 mmoL/L; TG: 0.83 ± 0.12 mmoL/L) (Figures 1B,D–F). In addition, serum HDL-C level that remained unchanged during aging, was significantly increased by LTDR (adults: 0.24 ± 0.04 mmoL/L; aged: 0.19 ± 0.03 mmoL/L; LTDR: 0.38 ± 0.05 mmoL/L) (Figure 1C). As it was expected, aged males had significantly lower levels of serum T, which were additionally decreased by LTDR (adults: 2.012 ± 0.24 ng/mL; aged: 0.46 ± 0.11 ng/mL; LTDR: 0.10 ± 0.019 ng/mL) (Figure 1G).

### 3.2 LTDR changes the expression of genes involved in *de novo* synthesis of CHOL and LDL-C metabolism in the liver

**Figure 2:**
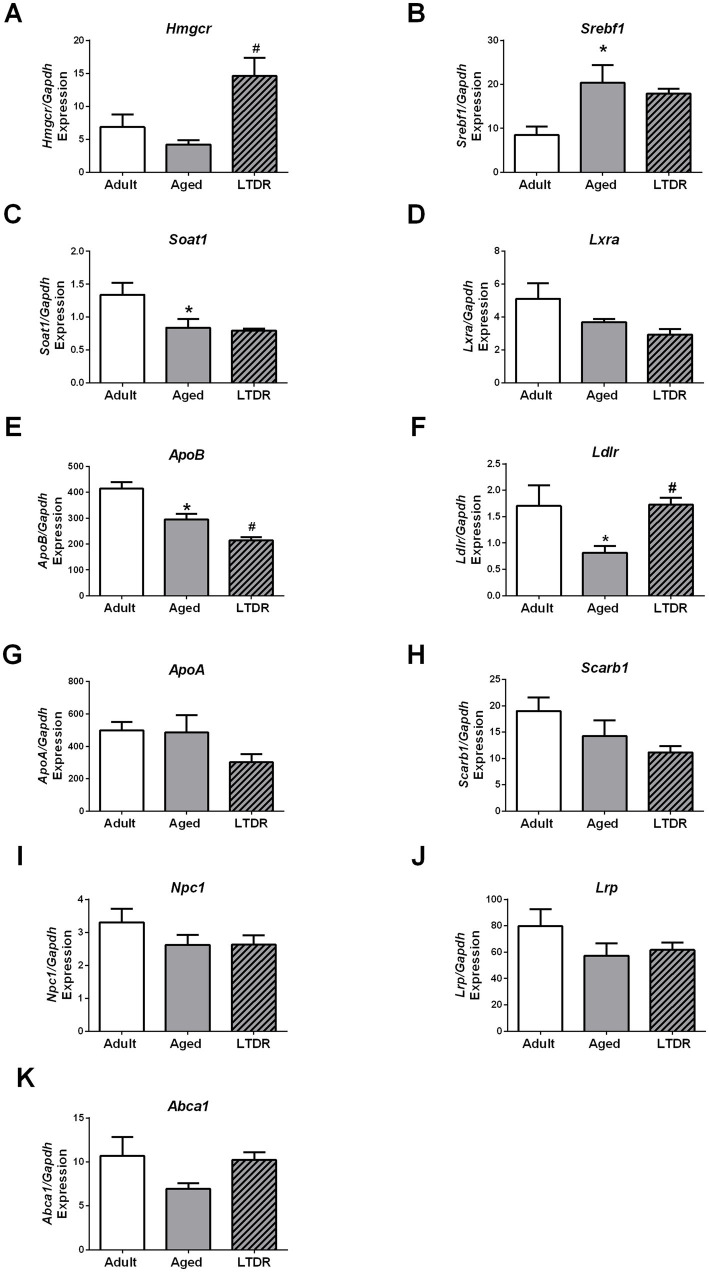
LTDR changed expression of the genes involved in lipid metabolism in the liver. To assess the effects of LTDR on gene expression, pieces of the livers from the adult, aged and LTDR groups were collected, total RNA was extracted, and qPCR was performed. Target gene expression (%) was determined by the comparative 2^−ΔΔCT^ quantification method with *Gapdh* as the reference gene. Time- and LTDR-induced changes in expression profile of *Hmgcr*, *Srebf1*, *Soat1*, *Lxra*, *ApoB*, *Ldlr*, *ApoA*, *Scarb1*, *Npc1*, *Lrp*, *Abca1* are presented at panels **A–K**. The bars shown are mean ± SEM values; for all the analysis *n* ≥ 5; ^*^indicates significant differences compared to adults; ^#^indicates significant differences between aged animals and the LTDR group; *p* < 0.05.

To get a better insight into the alterations of the lipid status, we investigated the expression profile of the genes involved in CHOL synthesis, esterification, and transportation. Expression of 3-hydroxy-3-methylglutaryl-CoA reductase (*Hmgcr*, gene for rate-controlling enzyme in CHOL synthesis) was unaffected by aging, but its expression level increased more than twofold following LTDR exposure (adults: 6.85 ± 1.89%; aged: 4.22 ± 0.66%; LTDR: 14.63 ± 2.71%) (Figure 2A). Age-induced gene expression was detected in the case of sterol regulatory element binding transcription factor 1 (*Srebf1*) (Figure 2B), while age-decreased expression was detected in the case of *Soat1* (Figure 2C). Compared to adults, trend of decreased expression of nuclear receptor subfamily 1 group H member 3 (*Lxra*) was recorded in aged animals without effect of LTDR (Figure 2D). Related to LDL mediated CHOL transportation and uptake, expression of *ApoB* was decreased in the aged group and additionally reduced after LTDR (Figure 2E). Expression of gene for LDL receptor (*Ldlr*) was decreased in aged animals, while LTDR reversed its expression to the levels detected in adults (adults: 1.7 ± 0.38%; aged: 0.8 ± 0.13%; LTDR: 1.73 ± 0.13%) (Figure 2F). Aging and LTDR did not affect the expression profiles of *ApoA* (Figure 2G) and *Scarb1*, both involved in HDL mediated CHOL transport and uptake, although a general trend of reduction was observed (Figure 2H). Changes were not detected in the expression profiles of *Npc1* and the gene for LDL receptor-related protein (*Lrp*) (Figures 2I,J) involved in intracellular trafficking, and *Abca1* responsible for CHOL efflux (Figure 2K).

### 3.3 LTDR-induced T decrement is not a consequence of disrupted pituitary function

**Figure 3:**
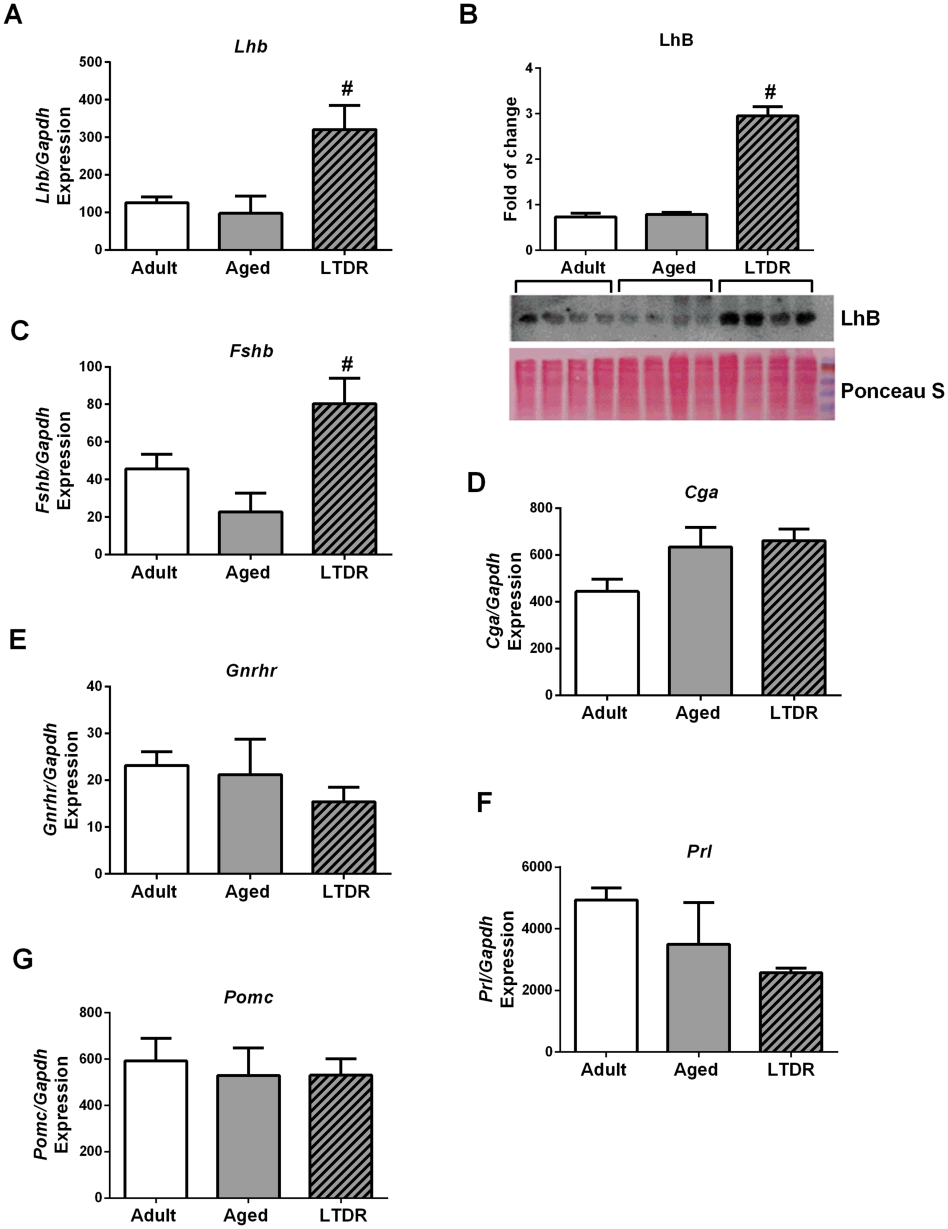
LTDR does not alter gonadotrophs function and elevated gonadotropins *b*-subunits expression in the pituitary. Pituitaries from all the groups were collected (*n* = 5), total RNA was extracted, and qPCR was performed. Target gene expression (%) was determined by the comparative 2^−ΔΔCT^ quantification method with *Gapdh* as the reference gene. Protein abundance was determined by western blot, relative fold of change was calculated based on the intensity of loading content and representative blot is shown. Panels show LTDR inducted expressions of *Lhb*/LhB **(A,B)** and *Fshb* **(C)**. And, expression profiles of *Cga* **(D)**, *Gnrhr* **(E)**, *Prl* **(F)** and *Pomc* **(G)** in advanced periods of life and after the LTDR regime. The bars shown are mean ± SEM values; ^*^indicates significant differences compared to adults; ^#^indicates significant differences between aged animals and the LTDR group; *p* < 0.05.

Since low T levels in advanced periods of life may be accompanied by changes in LH levels (13, 37), we evaluated if LTDR affected pituitary functionality and gonadotropin synthesis. Gene expression profile and protein abundance of *b* subunits (mediator of biological activity) of LhB were unchanged in advanced periods of life, however, LTDR drastically increased their expression (approximately 3-fold) (Figures 3A,B). Expression profile of *Fshb* was unaffected by aging, but approximately twofold increased after the LTDR regime (Figure 3C). Trends of increased expression of alpha polipeptide (*Cga*) (Figure 3D) and trends of decreased expression of *Gnrhr* (Figure 3E) and prolactin (*Prl*) (Figure 3F) were recorded in aged groups. The expression profile of proopiomelanocortin (*Pomc*) was stable in advanced periods of life (Figure 3G); LTDR had no effects on expression levels of *Cga*, *Gnrhr*, *Prl*, and *Pom*c. Presented results indicate preserved gonadotroph functionality under LTDR, and prompted us to focus our research on the functionality of testes and changed lipid homeostasis.

### 3.4 LTDR compromises testis capacity for androgen synthesis

**Figure 4:**
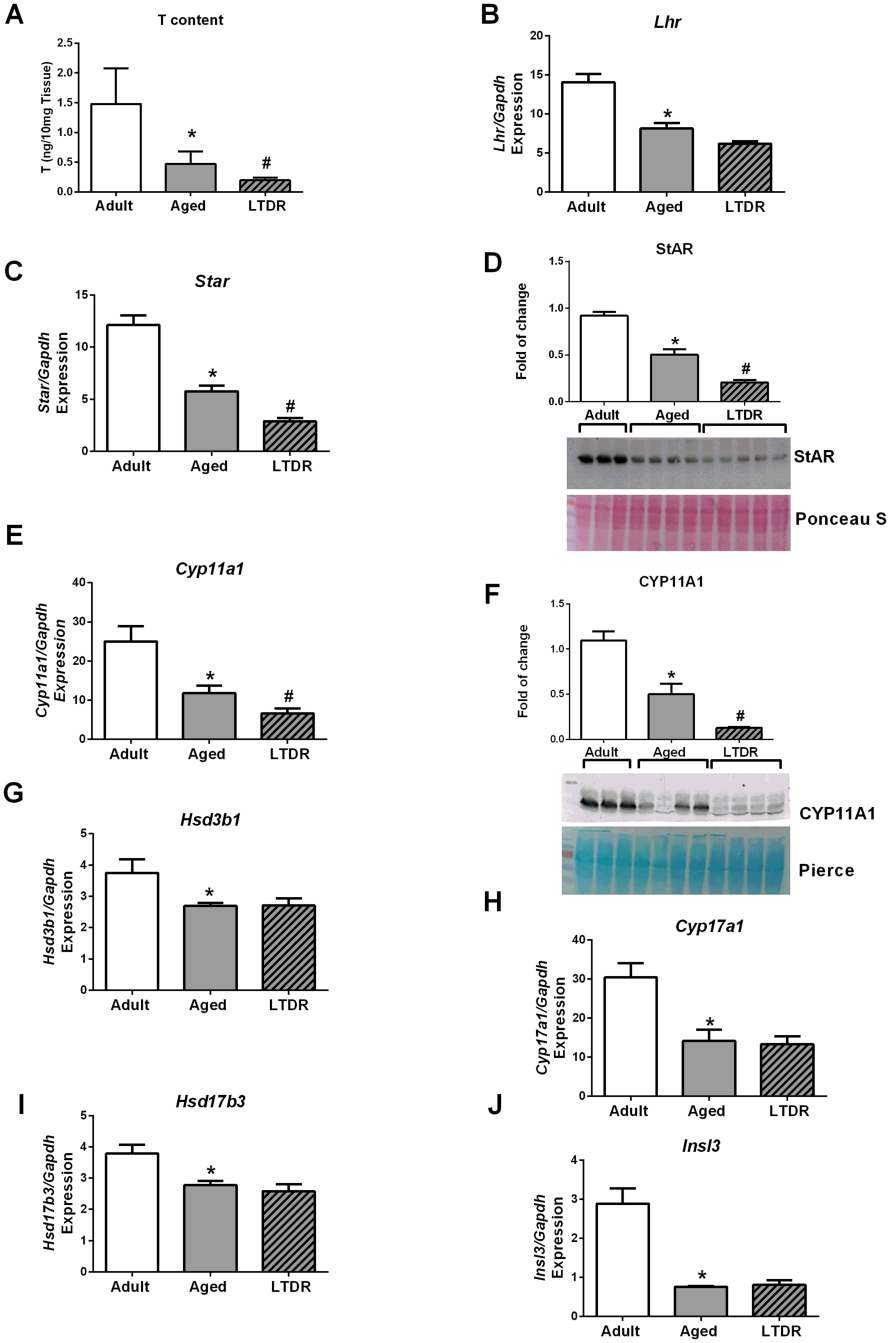
Effects of aging and LTDR on testicular androgen capacity and expression profile of components of testicular steroidogenic machinery. Pieces of testes (*n* = 5) were homogenized by 1 × PBS (10 μL/mg), extraction of steroids was done by ethyl-ether, dry pellet was resuspended in ELISA buffer and T content was determined by ELISA method **(A)**. Total RNA was extracted from pieces of testes (*n* = 5–6) and qPCR was performed. Expression profiles of *Lhr* **(B)**, *Star* **(C)**, *Cyp11a1* **(E)**, *Hsd3b1* **(G)**, *Cyp17a1* **(H)**, *Hsd17b3* **(I)** and *Insl3* **(J)** are shown. Protein abundance in the testes was determined by western blot, and representative blots of StAR **(D)** CYP11A1 **(F)** are shown. The bars shown are mean ± SEM values; ^*^indicates significant differences compared to adults; ^#^indicates significant differences between aged animals and the LTDR group; *p* < 0.05.

Following reduced serum T levels and preserved gonadotroph functionality after LTDR, we examined testes androgen capacity together with expression of key elements of steroidogenic machinery. According to obtained results, T content in the testes was significantly reduced in aged animals (adult: 1.48 ± 0.26 ng; aged: 0.47 ± 0.10 ng/10 mg tissue) and further decreased by LTDR (0.196 ± 0.02 ng) (Figure 4A). Compromised androgen capacity under LTDR was followed by unchanged expression of *Lhr* (Figure 4B) and reduced expression of *Star* (aged: 5.76 ± 0.54%; LTDR: 2.87 ± 0.34%) (Figure 4C) and *Cyp11a1* (aged: 11.74 ± 1.99%; LTDR: 6.59 ± 1.23%) (Figure 4E). Age and LTDR induced reduction in gene expression was confirmed by concomitant reduction in proteins (StAR Figure 4D) and (CYP11A1 Figure 4F) abundance. All the other examined genes, including *Hsd3b1* (Figure 4G), *Cyp17a1* (Figure 4H), *Hsd17b3* (Figure 4I), and *Insl3* (Figure 4J) showed age-dependent decrease in expression without significant impact of LTDR.

**Figure 5:**
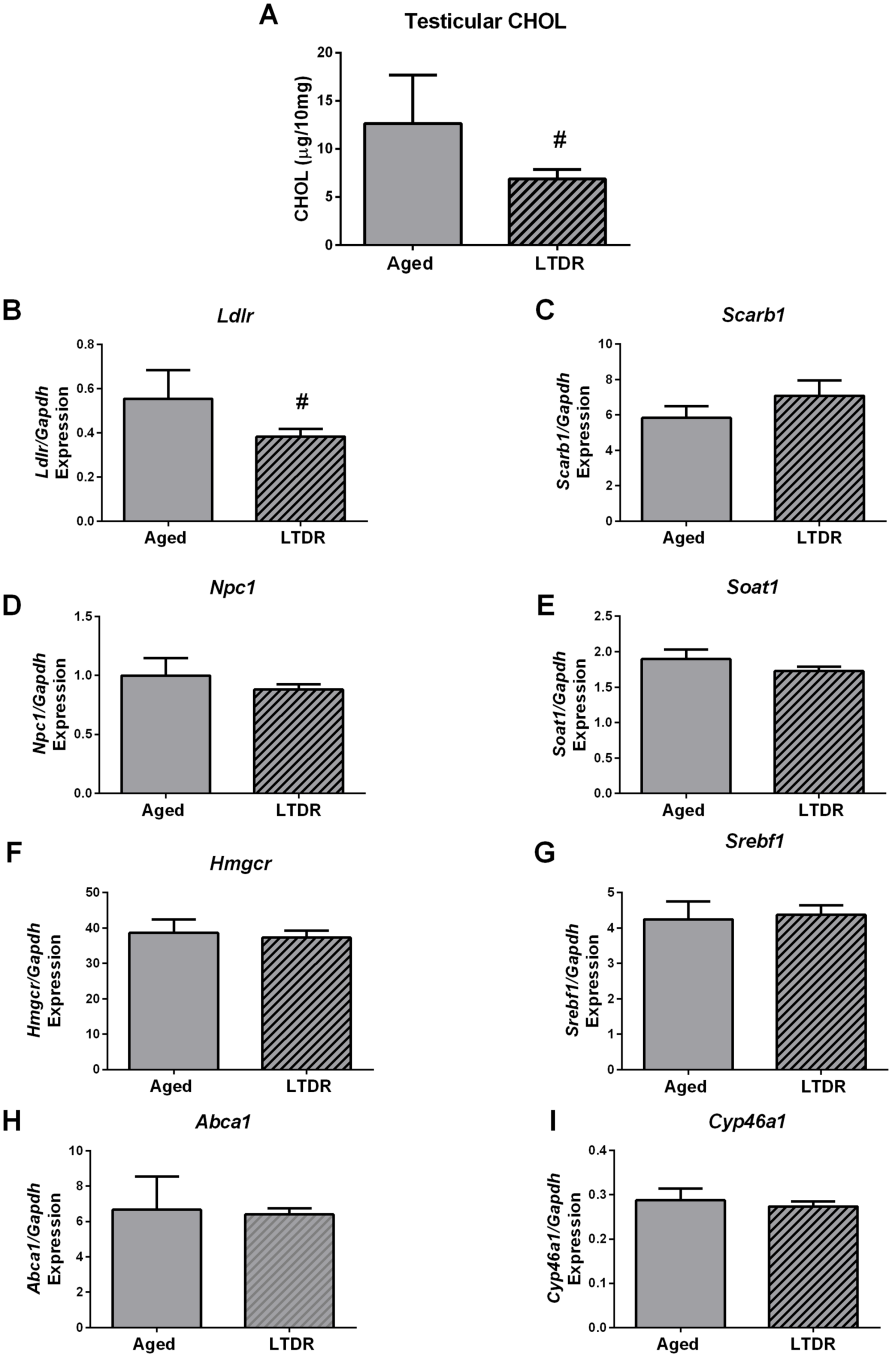
LTDR regime decreased *Ldlr* expression and CHOL in testes. Testicular tissue (*n* = 5–8) was homogenized with 1×PBS, extraction of lipids was done by ethyl-ether, dry pellet was resuspended in 0.1%-BSA-1×PBS, and CHOL content was photometrically measured **(A)**. Effects of LTDR on expression of the genes involved in cholesterol metabolism and *de novo* synthesis were assessed with qPCR method, *n* = 5 **(B–I)**. The bars shown are mean ± SEM values; ^#^indicates significant differences between aged animals and the LTDR group; *p* < 0.05.

To better understand processes that mediate LTDR-induced reduction in testes androgen capacity, we checked levels of CHOL in testes together with the expression profiles of genes involved in cholesterol metabolism. Indeed, testicular extracts from the LTDR group contained less CHOL than their *ad libitum* fed, age matched peers (aged: 12.64 ± 2.06; LTDR: 6.89 ± 0.99 μg/10 mg tissue) (Figure 5A), as well as decreased expression levels of *Ldlr* (aged: 0.55 ± 0.06%; LTDR: 0.38 ± 0.04; *p* = 0.038) (Figure 5B). Expression of other genes involved in maintaining CHOL homeostasis including *Scarb1*, *Npc1*, *Soat1*, *Hmgcr*, *Srebf1*, *Abca1*, and *Cyp46a1* was not affected by LTDR (Figures 5C–I).

### 3.5 Duration of DR as the main factor of the treatment effects

**Figure 6:**
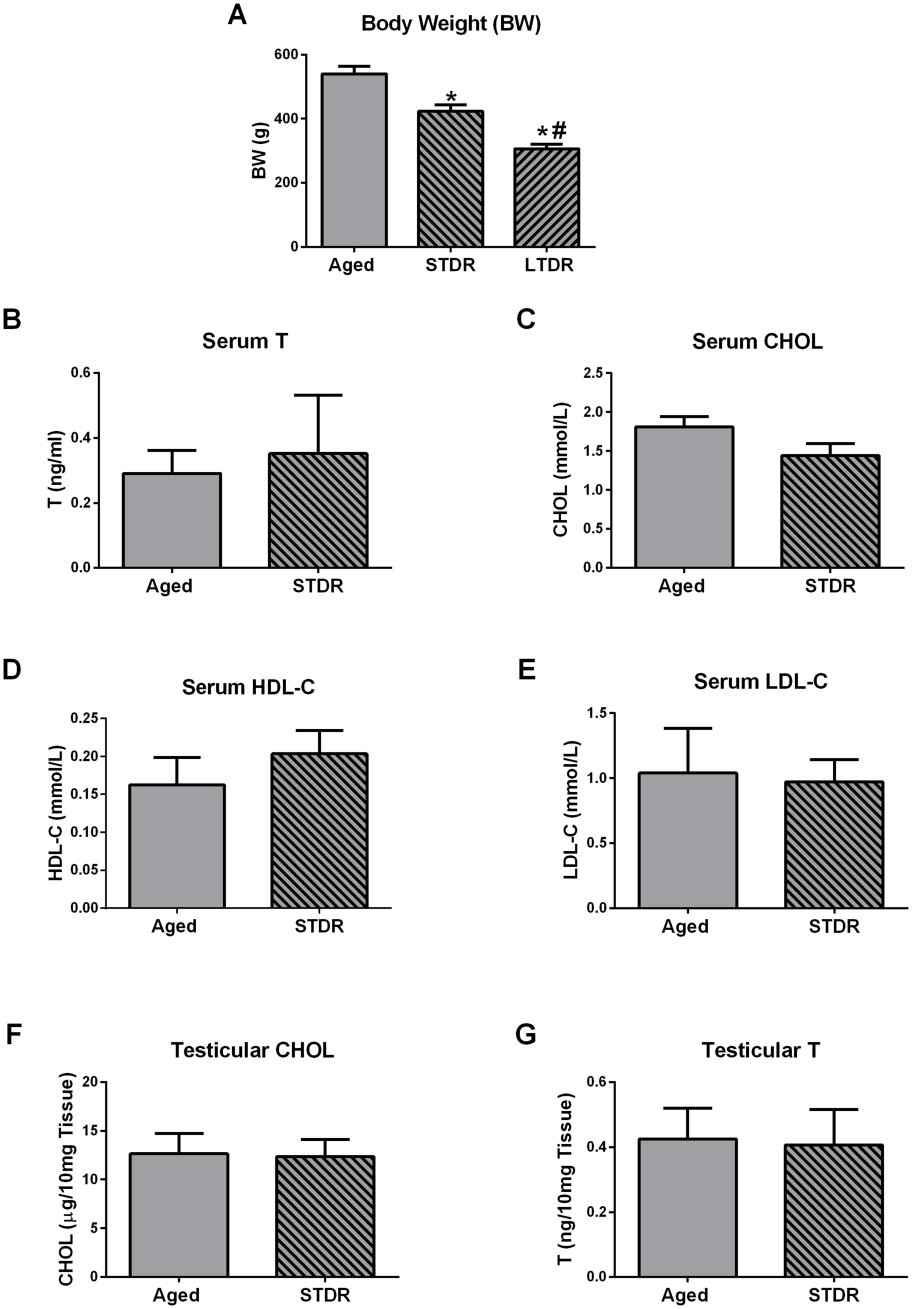
Short-term dietary restriction (STDR) changes BW but does not change T levels and lipid status in advanced periods of life. Aged male *Wistar* rats, males subjected to short-term dietary restriction (3-month-long, 60% of daily food intake, STDR), and males from LTDR group were monitored to estimate the effects of the regimes on the BW **(A)**. Serum levels of T **(B)**, CHOL **(C)**, HDL-C **(D)**, LDL-C **(E)**, as well as testicular levels of CHOL **(F)** and T **(G)** were determined. The bars shown are mean ± SEM values; for all the analysis *n* ≥ 5; ^*^indicates significant differences compared to aged animals; ^#^indicates significant differences compared to the STDR group; *p* < 0.05.

In the final steps of the study, we checked if the duration of the dietary restriction represents the crucial factor responsible for lipid status alterations and consequently a drop in T levels. To get an answer to this question, we exposed 21-month-old males to the 3-month-long 40%-dietary reduction (STDR) and inspected BW, T production, and lipid profile. Compared to the intact aged group, STDR decreased BW but with less effectiveness than LTDR (aged: 525 g ± 47.87 g; STDR 423.8 g ± 12.8 g; LTDR: 306.0 g ± 14.70 g) (Figure 6A). Serum levels of T, CHOL, HDL-C, and LDL-C did not differ significantly between aged and STDR animals (Figures 6B–E). In line with the presented findings, CHOL and T content in the testes of the STDR group were similar to the content detected in age-matched controls (Figures 6F,G).

## 4 Discussion

Aging is a complex process associated with the failure of multiple organ systems, including a decrease in T levels (14) and an altered lipid profile (20), both of which severely affect quality of life and correlate with a shorter lifespan and increased mortality (38, 39). Given that the elderly population is constantly growing (20), new treatments are needed for androgen deficiency and age-related perturbations in the lipidomic profile, suggesting lipid homeostasis disruption. In terms of reducing the amount of food without malnutrition, DR showed numerous beneficial effects, including normalizing total CHOL, LDL-C, and TG levels and increasing HDL-C levels (33). In obese and overweight men, DR significantly improved T levels, and weight loss was declared as the most appropriate intervention for obesity-related hypogonadism (40). Given the beneficial effects of DR on lipid status and T production, we wanted to investigate if those effects are related to similar changes in T production at advanced stages of life. We also wanted to investigate whether the duration of DR has any impact on those changes.

Here, we detected an increase in BW, serum CHOL, LDL-C, TG, and TG/HDL-C ratio in advanced stages of life without significant changes in HDL-C levels. LTDR effectively normalized all elevated parameters to the levels seen in adults, but also increased HDL-C levels. These results were expected and have been reported previously (33, 41, 42). The age-related increase in CHOL, LDL-C, and TG may be attributed to the increased expression of *Srebf1* observed in the liver, as SREBF1 acts as a key transcription factor that can induce lipogenesis and LDL synthesis (43, 44). Since it is known that HMGCR is a master regulator of CHOL *de novo* synthesis and that the expression of *Hmgcr* and *Ldlr* is controlled by a negative loop (45, 46), it is expected that increased expression of these genes will be detected following the LTDR-induced drop in circulating lipids. The drop in serum LDL-C was also accompanied by decreased expression of *ApoB*, and this finding is consistent with the previously published results (47); in the same publication, authors also detected an impaired TG synthesis and suppressed *de novo* fatty acid synthesis following DR. Decreased expression of *Lxra* in the liver has been documented in non-alcoholic fatty liver disease (48), often seen in the aged population, and a decreased pattern of *Soat1* might be related to dysregulation of cholesterol esters synthesis. Consequently, the results obtained on the expression of *Soat1* and *Lxra* may be suggestive of parameters indicative of disturbed CHOL homeostasis and hepatic functionality during the process of aging. Due to complex relations between changed lipid homeostasis and liver functionality at the latter stages of life, further investigations are needed to elucidate the exact mechanisms promoting age- and DR-induced changes in lipid status.

To check the possibility that LTDR impairs the pituitary function and consequently attenuates T production, the functional status of pituitary cells was examined. Based on the unchanged expression of *Cga*, *Fshb*, and *Lhb* we estimated preserved gonadotrophs functionality in advanced periods of life, which was proposed by earlier studies (16, 17, 49). Furthermore, the LTDR-induced increased expression of *Fshb* and *Lhb*/LhB, additionally indicates preserved responsiveness of gonadotrophs (37, 50), and most probably is a consequence of reduced T levels and activation of the testicular-pituitary feedback loop. Relatively stable expression of *Gnrhr* after LTDR, suggests that impaired GnRH signaling does not account for decreased T production. As it is known that hyperprolactinemia (51, 52) and increased activity of the pituitary-adrenal axis (53, 54) disturb gonadotrophs functionality and decrease T production, we inspected the expression profile of *Prl* and *Pomc*. Our results revealed a trend of decreased expression of *Prl* and unchanged expression of *Pomc* following LTDR, and based on these results, we excluded hyperprolactinemia and increased activity of the pituitary-adrenal axis as factors responsible for decreased T production. Considering that fasting or glucose deprivation suppresses pulsatile LH release (55), more detailed studies are needed, but at this stage, our findings have led us to focus our research on testicular functionality after LTDR.

Based on the results presented herein, it can be concluded that testicular functionality was significantly impaired during aging, reflected in decreased T content along with reduced expression of key elements of the steroidogenic machinery, including *Lhr*, *Star*/StAR, *Cyp11a1*/CYP11A1, *Hsd3b1*, *Cyp17a1*, *Hsd17b3*, and LCs functional marker *Insl3*. LTDR additionally decreased T content in the testes and the levels of StAR and CYP11A1. These results are in consistency with our previous work (15, 49) and relate to age-induced dysfunctionality of LCs rather than their loss (18, 50). The reduced androgen capacity induced by reduced food intake has been previously demonstrated in men (24) and rats (34–36). More recent studies showed a negative effect of caloric restriction on the testicular metabolome and sperm head formation in rats (56) and negative effects of dietary energy restriction on the testicular transcriptome in rams (57). To gain better insight into the mechanisms responsible for impaired T synthesis after LTDR, we analyzed testicular CHOL content with concurrent expression profiles of genes involved in CHOL synthesis and transport. Our results pointed to reduced CHOL content and decreased expression of *Ldlr* in the testes of the LTDR group, while the expression profiles of the other genes examined were comparable to those of age-matched controls. Unlike in liver, expression of *Hmgcr* stayed unchanged in testes and point to tissues specific regulation of *Hmgcr* expression and in *de novo synthesis* of CHOL. Difference in *Hmgcr* expression as well as unchanged expression of the genes involved in CHOL *de novo* synthesis and its uptake from HDL-C could be a consequence of impaired LH-cAMP signaling in aged LCs. This opinion comes from the fact that both process are controlled by LH-cAMP signaling in LCs (58) and not in liver. Since CHOL is an important precursor for testicular steroidogenesis, the impaired T production in the LTDR group could be a consequence of CHOL deficiency. Since it is known that CHOL needed for steroidogenesis is generated both through endogenously *de novo* synthesis and via circulating lipoproteins (1, 59), decreased *Ldlr* gene expression and serum LDL-C levels should be considered as possible cause. Due to the extremely complex regulation of testicular lipid homeostasis, it is difficult to determine the exact source of cholesterol used for testosterone synthesis (1). HDL-C has been proposed as the major extracellular source of CHOL for LCs, while LDL-C is also known to support testicular steroidogenesis under certain conditions such as desensitization promoted by hCG/LH (60). It is also known that rat HDL contains apolipoprotein E and has a high affinity for LDLR, and presence of HDL with apolipoprotein E increases the T production of rat LCs (61). *In vivo* studies showed that simvastatin-treated rats had lower T and LDL-C levels (62), while Klinefelter et al. (63) demonstrated a statin-induced reduction in LH-stimulated T production in rat LCs. Population studies also showed a positive association between lower LDL-C levels and free T levels (64) and a reduction in total T, free T, and LDL-C after statin use (65). In the context of impaired LDLR signaling, it has been shown that *ApoE*^−/−^*Ldlr*^−/−^ mice exhibited reduced T levels, sperm counts, and testicular tubule atrophy (66). It has also been shown that low T levels promote the reduction of LDLR and decreased uptake of CHOL in the liver of rats (67). Considering that we did not use purified LCs in this study, further mechanistic studies are needed to clarify the role of LDL-C in T production under LTDR. Since LDL-C is known to play a role in the development of atherosclerosis and consequently in inflammation, we excluded the possibility that inflammation in the testis is responsible for LTDR-promoted T reduction based on the expression profile of inflammatory cytokines (Supplementary Figure 1). Based on serum levels of corticosteroids and the expression of *Nr3c1* and *Hsd11b1/2*, we excluded the involvement of stress in LTDR-mediated T decline (Supplementary Figure 2); non-elevated corticosterone levels are consistent with unchanged levels of pituitary expression of *Pomc* in the LTDR group. In contrast to the effects of LTDR, STDR reduced body weight, but to a lesser extent than LTDR, and had no adverse effects on serum and testicular T levels. The treatment also did not alter serum CHOL, HDL-C, LDL-C, and testicular CHOL levels.

In consideration of the results that have been presented, it can be concluded that LTDR, but not STDR, was able to reduce T production at advanced stages of life. Reduced testosterone levels may be a consequence of testicular CHOL deficiency, due to reduced serum CHOL and LDL-C levels after LTDR. The findings reported in this study indicate that the duration of restricted food intake is an essential factor responsible for alterations in lipid homeostasis and compromised androgen capacity of testes. In accordance with the results of this study, which demonstrate the adverse effects of LTDR, it is imperative that individuals practice extreme caution when considering its implementation in the advanced stage of life.

## Data Availability

The original contributions presented in the study are publicly available. This data can be found in the institutional repository: https://radar.ibiss.bg.ac.rs/.

## Footnotes

^1^ www.biosystems.global

^2^ www.invitrogen.com

^3^ www.fishersci.com

## Supplementary material

SUPPLEMENTARY FIGURE 1 LTDR does not trigger testicular inflammation and the inflammation-related T reduction. Total RNA was extracted from pieces of testes (*n* = 5–6), and qPCR was performed. Target gene expression (%) was determined by the comparative 2^−ΔΔCT^ quantification method with *Gapdh* as the reference gene. Expression profiles of *Nos2* **(A)**, *Lif* **(B)**, *Il1b* **(C)**, *Il2* **(D)**, *Il6* **(E)**, *Il10* **(F)**, *Tnf* **(G)**, and *Hif1a* **(H)** are shown. The bars shown are mean ± SEM values; ^*^indicates significant differences compared to adults; ^#^indicates significant differences between aged animals and the LTDR group; *p* < 0.05.

SUPPLEMENTARY FIGURE 2 Corticosterone and corticosterone-related signaling do not participate in LTDR-induced T reduction. Serum corticosterone was determined by Enzo Corticosterone ELISA kit **(A)**. Total RNA was extracted from pieces of testes, and qPCR was performed. Expression profiles of *Nr3c1* **(B)**, *Hsd11b1* **(C),** and *Hsd11b2* **(D)** are shown. The bars shown are mean ± SEM values; for all the analysis *n* = 5–6; ^*^indicates significant differences compared to adults; ^#^indicates significant differences between aged animals and the LTDR group; *p* < 0.05.
