# Supplementary material for: Long-term dietary restriction changes lipid homeostasis and consequently impairs testosterone production in aged Wistar rats
Source: Front Nutr. 2025 Sep 19;12:1665682. doi: 10.3389/fnut.2025.1665682 (PMC12490992; doi:10.3389/fnut.2025.1665682)
Supplement: Supplementary file 3 [file Table_1.pdf]

Supplement Table 1. Pairs of primers used for qPCR

Gene	Primer sequence (5' to 3')	Accession Number
Apoa1	F: 5' TCTTCCTGACAGGTTGCCAAG<br>R: TGGCGAAATCCTTCACCCTG	NM_012738.2
Cga	F: TAGCTTCCATGTGTGCCAAG<br>R: GTGACAGGAAAGCAGCAGTG	NM_053918.2
Cyp11a1	F: ACTTCCTGAGGGAGAACGGC<br>R: TCCATGTTGCCCAGCTTCTC	NM_017286.3
Cyp17a1	F: GTTTCTCCCCAGACGTGGTC<br>R: GGTCCGACAAGAGGCTTTGA	NM_012753.2
Fshb	F: CACTGCCTTAGGGACCATCG<br>R: CCCCAAGCTGTGAGTTGAGA	NM_001007597.2
Gnrhr	F: CTGCCTTCAATGCCTCTTTC<br>R: AGCGGCATGACGATTAGAGT	NM_031038.3
Hif1a	F: TCAAGTCAGCAACGTGGAAG<br>R: TATCGAGGCTGTGTCGACTG	NM_024359.2
Hsd11b1	F: AAATACCTCCTCCCCGTCCT<br>R: TTTCTCTTCCGATCCCTTTG	NM_017080.2
Hsd11b2	F: CAAACCCTTCCCCCACAG<br>R: GGCTGGGCTTTTCTTAACAG	NM_017081.2
Hsd17b3	F: CTGCTTGTGTGCCTCGTTTG<br>R: ACTGCCCATTGTCCCATTGA	NM_054007.1
Hsd3b1/2	F: GACAGGAGCAGGAGGGTTTGTGG<br>R: CTCCTTCTAACATTGTCACCTTGGCCT	NM_001007719
Il1b	F: AAACAGCAATGGTCGGGACA<br>R: GTCCTGGGGAAGGCATTAGG	NM_031512.2
Il2	F: GCTTTCACTTGGAAGACGCTG<br>R: GGCTCATCATCGAATTGGCA	NM_053836.1
Il6	F: GCCCACCAGGAACGAAAGT<br>R: GGCAACTGGCTGGAAGTCTC	NM_012589.2
Il10	F: GCTCAGCACTGCTATGTTGC<br>R: GTCTGGCTGACTGGGAAGTG	NM_012854.2
Insl3	F: TACTGCTGCTCCTGGCTCTA<br>R: GCAGCAGCTCCCGGTC	NM_053680.1
Lhb	F: AGCATGGTTCGAGTACTGCC<br>R: GACCCCCACAGTCAGAGCTA	NM_001033975.1
Lhr (Lhcgr)	F: TATGCTCGGAGGATGGCTCT<br>R: AGCACAGATGACGACGAAGG	NM_012978.1
Lif	F: GTGTCCCCGACAACTCTAGC<br>R: GGACCACCGCACTAATGACT	NM_022196.2
Lxra	F: TCAGCATCTTCTCTGCAGACCGG<br>R: TCATTAGCATCCGTGGGAACA	XM_008762013.1
Nos2	F: CAGCCCTCAGAGTACAACGAT<br>R: CAGCAGGCACACGCAATGAT	NM_012611.3
Nr3c1 (Gr)	F: TCTCAGGCAGATTCCAAGCA<br>R: TGGACAGTGAAACGGCTTTG	XM_039096567.1

Pomc	F: AGAACGCCATCATCAAGAACG<br>R: AGGTCAGGTGCTCTCGCC	NM_139326.2
Prl	F: GGGGAAGAGGATGCCTGATT<br>R: GGAGTGTCCCTGCTTTCCG	NM_012629.1
Prlr	F: GGATTTGATACCCATCTGCTG<br>R: CCAGCAAGTCCTCACAGTCA	NM_001034111.1
Scarbf1	F: GCTTCTGGTGCCCATCATTTAC<br>R: AGCTTGGCTTCTTGCAGTACC	NM_031541.1
Star	F: AGCAAGGAGAGGAAGCTATGC<br>R: GGCACCACCTTACTTAGCACT	NM_031558.3
Tnf	F: CCCCCATTACTCTGACCCCT<br>R: CCCAGAGCCACAATTCCCTT	NM_012675.3
